# Food and medicinal uses of *Annona senegalensis* Pers.: a country-wide assessment of traditional theoretical knowledge and actual uses in Benin, West Africa

**DOI:** 10.1186/s13002-022-00510-2

**Published:** 2022-03-04

**Authors:** Janine C. F. Donhouedé, Kolawolé Valère Salako, Kisito Gandji, Rodrigue Idohou, Roméo Tohoun, Achille Hounkpèvi, Natasha Ribeiro, Ana I. Ribeiro-Barros, Romain Glèlè Kakaï, Achille Ephrem Assogbadjo

**Affiliations:** 1 Department of Forest Engineering, Faculty of Agronomy and Forest Engineering, Eduardo Mondlane University, P.O. Box: 257, Maputo, Mozambique; 2 Laboratoire de Biomathématiques et d’Estimations Forestières, Faculty of Agronomic Sciences, University of Abomey-Calavi, 04 BP: 1525 Cotonou, Benin; 3 Ecole de Gestion et de Production Végétale et Semencière, National University of Agriculture, Kétou, Benin; 4 Plant Stress & Biodiversity Lab-Forest Research Center (CEF), School of Agriculture, University of Lisbon, 1349-017 Lisbon, Portugal

**Keywords:** Use patterns, Knowledge gap, Sociolinguistic groups, Socio-demographic factors, *Annona senegalensis*

## Abstract

**Background:**

The growing interest for more natural products in food and health industries has led to increasing research on traditional knowledge related to plants. While theoretical knowledge (TK) on the uses of a species informs on the wide spectrum of potential uses of that species, actual uses (AU) highlight their potential being actually used. Distinguishing between the two is important when reporting ethnobotanical studies. However, studies often equated AU and TK, sometimes misleading conclusions, and decision-making. This study assessed TK, AU, and difference between TK and AU of *Annona senegalensis* and how each is related to factors such as age, sex, sociolinguistic group, and main activity in Benin republic.

**Methods:**

Data were collected through semi-structured individual interviews (*n* = 755) and analyzed using among others, relative frequency of citation (RFC), and use-value (UV).

**Results:**

A total of 168 theoretical uses were recorded but only 92 were “actually” practiced, of which four were food and 88 medicinal uses. TK and AU were positively correlated. As expected, TK was also significantly higher than AU, indicating that some potential uses of the species are still not valued. Sociolinguistic group and main activity, not age and sex, were the main factors influencing TK, AU, and difference between TK and AU. The highest TK was found with Bariba sociolinguistic group and the highest AU with Otamari. Fruits (100%) and flowers (10%) were the most used organs for food, while leaves (40%) and roots (7%) were mostly used for medicinal purposes. The most common food uses were consumption of the ripe fruits (100%), and food seasoning with flowers (10%). The most cited diseases were malaria (28%) and intestinal worms (8%).

**Conclusions:**

The study illustrated the importance of differentiating between TK and AU. It documented  the wide range of the uses of *A. senegalensis*, while highlighting its most common uses, and the need to better valorize and sustainably manage the species.

**Supplementary Information:**

The online version contains supplementary material available at 10.1186/s13002-022-00510-2.

## Background

Ethnobotany is a multidisciplinary science aiming at understanding the relationship between humans and plants [1]. Understanding how and why people select and use some plants can help improving their conditions while sustainably managing biodiversity [2]. Ethnobotanical knowledge is therefore essential for the assessment, valorization, and sustainable management of natural resources [3, 4]. Several ethnobotanical surveys have reported the importance of plants, showing generally the most popular food and/or medicinal plants, plant parts used, and the different diseases healed [5–7]. However, the methods often used in ethnobotanical studies hardly distinguish between the knowledge and the actual use of the plant species [8].

Theoretical knowledge (TK) refers to the different information acquired through formal and/or informal instruction about the ethnobotanical uses of plants, while actual use (AU) is what has been really experienced or practiced [8, 9]. AU is therefore expected to be less or equal to TK. AU would be equal to TK if all known uses are practiced. While TK could help to know the potential of a given species, AU allows to ascertain the current actual importance, the preferences and to some extent the threats to the species [9]. Both TK and AU are essential to understand the importance of a species and its future potential to enhance human’s livelihoods.

The few available studies that focused on the difference between TK and AU have considered knowledge and uses of multiple plants, where the variables analyzed were the number of known plants and the number of plants actually used [8, 10]. Very few have considered single species [11], bringing together the number of known uses and the number of practiced uses. In either case, if the relationship between TK and AU is positive and strong, TK could be considered a good proxy of AU, and using any of the two should provide the same patterns. The more knowledge one has on a species, the greater the use of that species. However, previous studies showed that the relationship is not always positive and sometimes could rather be neutral [8, 10]. For instance, in a study in Bolivian Amazon, Reyes-García et al. [8], found a positive relationship between TK and AU in an isolated village, but no relationship in a non-isolated village where people are less dependent on forest resources. The author argued that when indigenous people become more integrated into the market economy and adopt plant substitutes, they stop using plants, which dilutes the relationship between TK an AU.

Further, distinguishing between TK and AU matters in several instances. The gap between TK and AU could have substantial conservation implications. For example, Ahoyo et al. [12] calculated species use-value as an indicator of the intensity of uses, i.e., threats to the species. In this case, not distinguishing between AU and TK when calculating species use-value could lead to erroneous assessment of the intensity of uses. Also, de Luceana et al. [9] showed that to test the ecological appearance hypothesis which predicts that *the most available species are the most often used*, assessing species use-value based on TK or AU might not lead to the same conclusion. The gap between TK and AU has often been interpreted in terms of knowledge erosion [13]. Yet, this interpretation might not be applicable in some circumstances. de Albuquerque et al. [13] rather proposes two concepts “mass knowledge” and “stock knowledge.”

Some authors reported that the gap between TK and AU might be related to the replacement of some tree species by the more available, rapid socioeconomic change and/or erosion of knowledge [8, 10]. de Albuquerque et al. [13] further interpreted this gap as a diversification of knowledge rather than erosion of knowledge and state that species which are known and less used might only be used by communities when needed specifically.

As illustrated above, more data are still needed to better understand the relationship between TK and AU, as well as the difference between the two. As suggested by Reyes Garcia et al. [8], the difference between TK and AU could be linked to the socio-demographic context. For instance, one would expect that the gap between TK and AU is related to socio-demographic factors such as sex (men *versus* women), age (young *versus* adult), sociocultural group (ethnic groups), and socio-professional categories (e.g., farmers *versus* non-farmers, traditional healers *versus* non-traditional healers). Understanding how the gap between TK and AU is related to the above factors might improve our knowledge of the dynamics of traditional knowledge and uses of plants, and the consequences for biodiversity conservation.

**Fig. 1:**
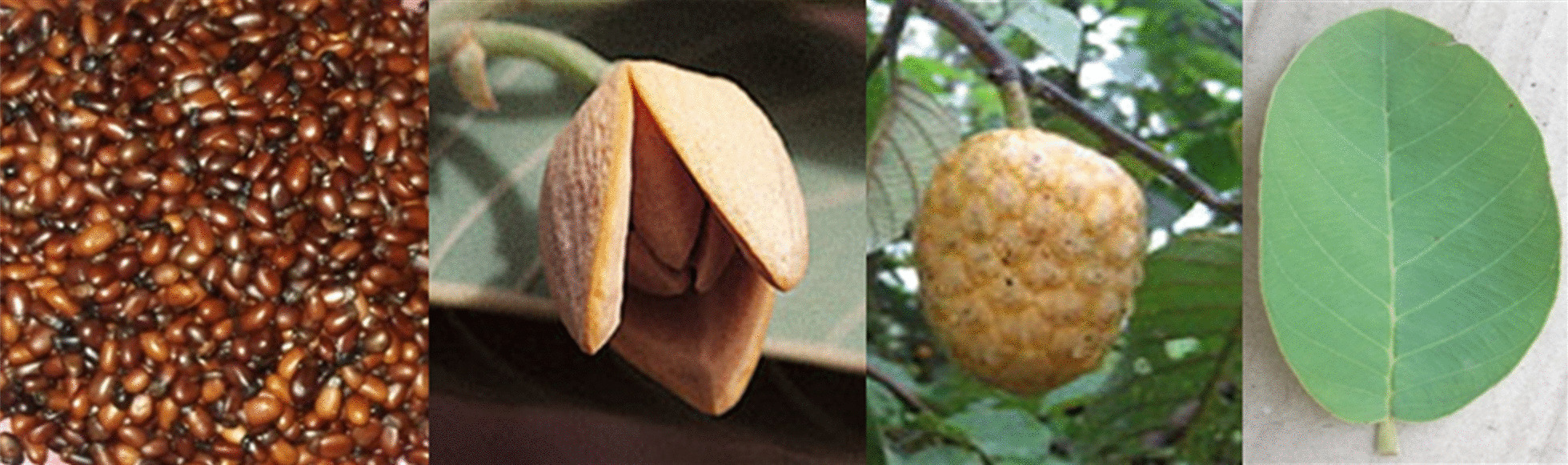
Seeds, flower, fruit, and leaf (from left to right) of *A. senegalensis*

This study focused on the wild custard apple, *Annona senegalensis* Pers., particularly its food and medicinal uses because these are among the most important basic needs of human livelihoods [14]. The wild custard apple is a multipurpose shrub, 2 to 6 m tall that can reach 11 m height under favorable conditions [15]. The fruit is formed from several fused, freshly, and ovate carpels of about 45 mm in diameter (Fig. 1). At early development, the fruit is dark green repining to yellow and finally to orange when ripen [15]. In Africa, many authors have reported on the importance of *A. senegalensis* as a food and medicinal plant. Okhale et al. [16] reported that different parts of the species are used in traditional medicine to treat several diseases including tuberculosis, hernia, diabetes, gastritis, male sexual impotence, difficulty in swallowing and snake bites. The potential of the species in the management of a minimum of three COVID 19 symptoms such as cough, fever, myalgia, and the treatment of several types of cancer (liver, breast, and colon cancer) has also been reported [17, 18]. Regarding food uses, the fruit of *A. senegalensis* has a sweet taste and is highly appreciated. The flowers and the seeds with aromatic flavor are used by indigenous population to season food [19]. *A. senegalensis* is also of major commercial importance, contributing significantly to household income [20–22]. In Benin, the species is collected in the bush, from fallows, savannas and often cut down for crop production. More so, the ongoing change in land uses and land cover added to climate change are major threats to the species which call for its sustainable management and conservation [23].

There are several reports that knowledge and uses of tree species are associated among others to socio-demographic, socioeconomic and sociocultural factors. Among these, age, sex, main activities [24, 25] and sociolinguistic group [26, 27] were frequently found to have an influence on ethnobotanical knowledge and use patterns of tree species. In this study, we asked the following questions: what knowledge does local people have on the uses of *A. senegalensis*? which of the known uses are practiced in reality? To what extent do both differ? How far do age, sex, sociolinguistic group, and main activity determine TK, AU, and gap between TK and AU of *A. senegalensis*? To answer these questions, we tested the prediction that the use-value calculated based on TK and the one calculated based on AU are positively correlated. We also tested the prediction that the use-value calculated based on TK would be different from the use-value calculated based on AU, and that both use-values and their difference would vary with informants age, sex, sociolinguistic group, and main activity.

## Materials and methods

### Study area

**Fig. 2:**
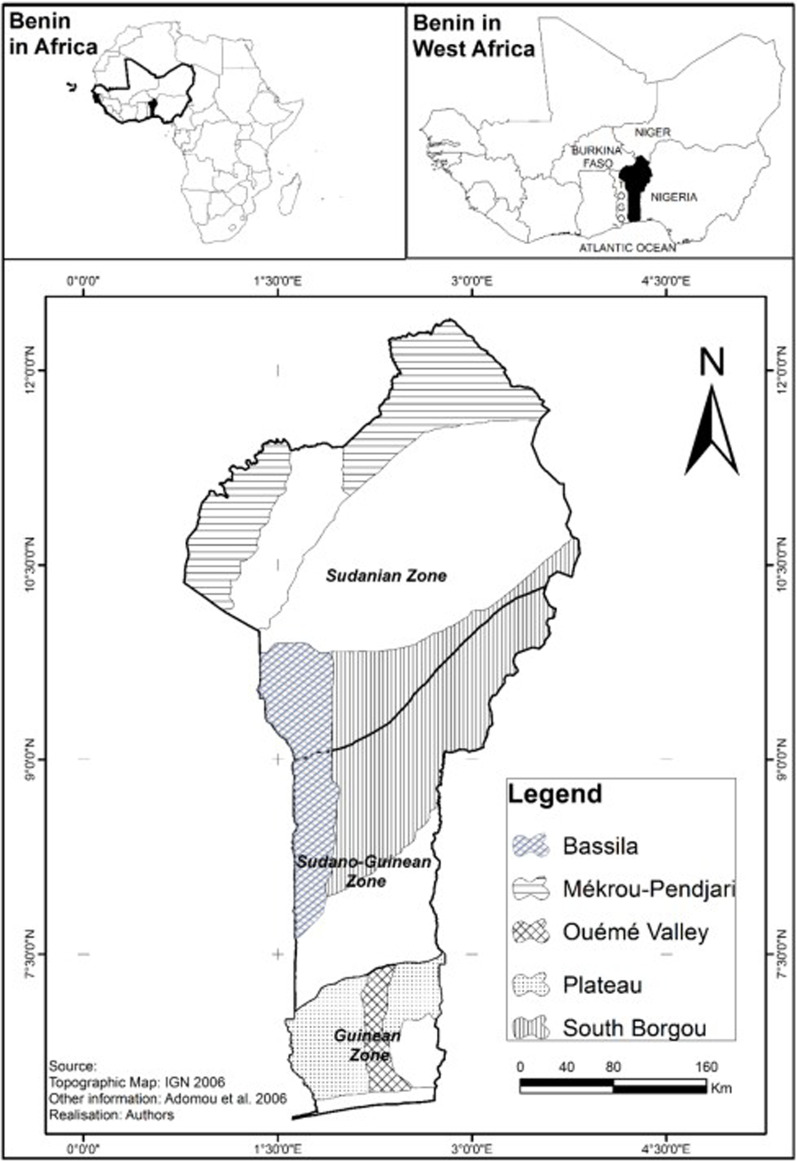
Geographical location of the study area

This study was conducted in five phytogeographical districts in the Republic of Benin. Located in West Africa (6°12°50′ N and 1° 3°40′ E), Benin covers an area of 114,763 km^2^ with a population over 11 496 140 inhabitants [28]. The climate is generally dry, composed of the subequatorial Guinean region (6°25ʹ-7°30ʹN), the Sudano-Guinean region (7°30ʹ-9°30ʹN) and the Sudanian region (9°30ʹ-12° N). The vegetation is composed of dry dense forests, mosaic of woodlands, savannas, and gallery forests. Data were collected in five of the ten phytogeographical districts of the country (Fig. 2). The South-Borgou, Bassila (Sudano-Guinean region), Mekrou-Pendjari (Sudanian region), Oueme-valley and Plateau (Guinean region) correspond to the most important areas in terms of abundance of *A. senegalensis* [29]. The sociolinguistic groups found in the study area are Bariba, Beyonbe, Dendi, Holli, Idaasha, Kountema, Lokpa, Mahi, Nago, Natimba, Otamari, Tankama, Wémènou and Yom.

### Sampling procedure and data collection

This study was carried out from November 2019 to March 2020. Data were collected through individual interviews using a semi-structured questionnaire. The five phytogeographical districts where the species more naturally occurs in Benin were considered [30]. In each phytogeographical district, we randomly selected 151 informants and ensure representativeness of the informants considering sociolinguistic group, main activity, sex, and age. This number was determined through the normal approximation of the binomial distribution [31]:

$$n = \frac{{U_{1 - \alpha /2}^{2} \times p\left( {1 - p} \right)}}{{d^{2} }}$$

where* n* is the sample size considered in the phytogeographical district; *U*^*2*^_1−*α/2*_ is the random normal variable value for a probability of *α* = 0.05; *U*^2^_1−*α*/2_ = 3.84; d is the margin of error set at 0.08; *p* is the proportion of informants who know the species. We considered the maximum possible sampling size, i.e., considering *p* = 0.5. In total, 755 informants (Table 1) were individually interviewed.

**Table 1:** Distribution and variation in the number of respondents according to their characteristics

Characteristics	Modality	Number of respondents	Relative frequency
Gender	Woman	287	38.52
	Man	468	61.81
Age	Young (age ≤ 35)	133	17.85
	Adult (35 < age ≤ 60)	433	58.12
	Old (age > 60)	189	25.36
Main activity	Farmer	354	47.51
	No_Farmer	378	50.73
	Trad_healers	23	3.08
Sociolinguistic group	Bariba	100	13.42
	Beyonbe	25	3.36
	Dendi	50	6.71
	Idaasha	19	2.55
	Kountema	25	3.31
	Lokpa	50	6.71
	Mahi	65	8.72
	Nago	81	10.87
	Natimba	51	6.85
	Otamari_	19	2.55
	Berba	13	1.74
	Tankama	50	6.71
	Weme	146	19.6
	Yom	51	6.85

Data collected using the questionnaire were related to informants’ biodata (name, age, sex, sociolinguistic group and main activity). All known food and medicinal uses of the species by the informants were recorded as theoretical knowledge (TK). For each known use, the informant was asked whether he/she practices; these uses were considered as actual uses (AU). The preparation methods, additives/ingredients used during preparation, administration methods and doses, and the perceived effectiveness were also recorded for each use of the species. The perceived effectiveness was scored for each experienced medicinal use using a three-level Likert scale: “not effective” (coded 1), “effective” (coded 2), and “strongly effective” (coded 3).

### Data analysis

#### Diversity of food and medicinal uses of *A. senegalensis* and relationships with sociolinguistic groups

All the known uses of *A. senegalensis* reported during the study were summarized in table. For each specific use, the plant part involved, the method of preparation, the administration mode, the dosage, and the average effectiveness score were summarized. The Relative Frequency of Citation (RFC) was calculated for each specific food and medicinal use. The RFC is a measure of informant consensus on a specific use and is calculated as follow [26]:

1 $$RFC_{u} = \, FC_{u} /N$$

where *FC*_*u*_ is the number of informants who mentioned the specific use (*u*), *N* is the total number of informants surveyed.

Sankey diagrams were established to illustrate the association between sociolinguistic groups and the food and medicinal specific uses. The RFC of each plant part for either food or medicinal uses was also calculated to determine which plant part was the most solicited. Because medicinal uses were the most diverse, further analyses were carried out to (i) assess the links among medicinal uses and (ii) determine association between medicinal uses and the sociocultural groups. The aim was to determine the convergent medicinal uses and non-convergent uses. For this purpose, the RFC of the actual use of each specific medicinal use was computed per sociocultural group, and the obtained matrix was submitted to a principal component analysis (PCA).

#### Factors influencing traditional theoretical knowledge (TK), actual uses (AU) and gap between TK and AU

Descriptive statistics (min, max, and median) were first calculated for TK, AU, and the difference between TK and AU. Then, the scatterplot of TK and AU was established, and Pearson correlation at the significance level 0.05 was used to test the direction and significance of the relationship between TK and AU.

The use-value was used to calculate TK and AU values for the sample. The use-value (UV) is calculated as follow [9]:

2 $$UV = \frac{{\mathop \sum \nolimits_{i = 1}^{n} u_{i} }}{n}$$

In Eq. 2*u*_*i*_ is the number of uses reported by informant *i*, and *n* is the total number of informants. When *u*_*i*_ is taken as the number of all known uses of *A. senegalensis* cited by the informant *i*, UV is equivalent to TK. When *u*_*i*_ is taken as the number of uses of *A. senegalensis* practiced by the informant *i*, UV is equivalent to AU.

TK and AU were calculated irrespective of the use-categories, i.e., TK_Total_, and AU_Total_. The gap between TK and AU was calculated as the difference TK_Total_–AU_Total_. AU was further calculated per use-category, i.e., AU_Food_ for food use-category, and AU_Medicinal_ for medicinal use-category. AU_Food_ and AU_Medicinal_ were calculated to assess the food and medicinal use-value for the studied communities. Poisson generalized linear model was used to test the effects of informants’ age, sex, sociolinguistic group and main activity group on TK_Total_, AU_Total_, TK_Total_–AU_Total_, AU_Food_, and AU _Medicinal_.

The full model, i.e., the one including all main effects and possible interactions was first established. The parsimonious model was then determined using a backward elimination based on the corrected Akaike Information Criteria (AICc).

All analyses were conducted in R software version 3.5.1 (R Core Team 2018). The principal component analysis (PCA) was performed using the package *FactoMineR* [32].

## Results

### Diversity of uses of *A. senegalensis* and relationships with sociolinguistic groups

**Fig. 3:**
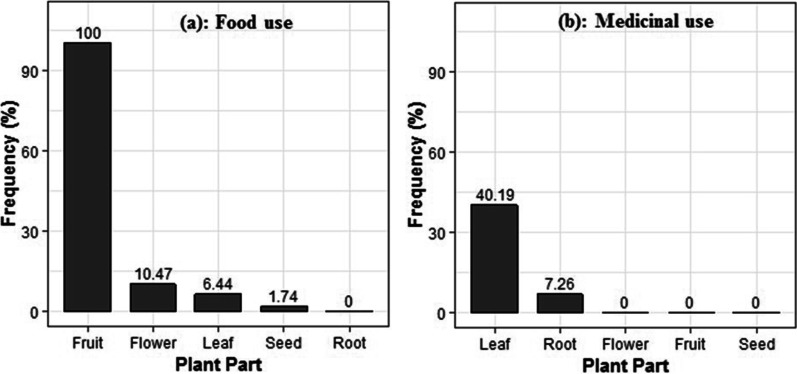
Plant parts used according to use-categories

A total of 168 theoretical uses were recorded for *A. senegalensis* but only 92 were effectively practiced, of which four were food and 88 medicinal uses. Among the practiced uses, four food and fourteen medicinal uses with RFC ≥ 1% were reported for *A. senegalensis*, of which two and nine, respectively, had a RFC equal or greater than 2% (Table 2). Fresh fruits are eaten ripe (100%), leaves (6.44%) and flowers (10.47%) are used to prepare sauce, and seeds (1.74%) are used for sauce seasoning (Fig. 3a). Among the fourteen medicinal uses, the use of leaves to treat malaria anddysentery, and the use of roots for snake bites had the highest RFC. On average, all specific uses experienced by informants were effective (Table 2).

**Table 2:** Diversity of medicinal uses of *A. senegalensis* and perceived effectiveness – Only uses with RFC ≥ 1% are listed in this table

Plant part	Specific uses	Method of preparation	Administration	Dosage	RFC (%)		Average effectiveness
					Overall	Actual	
Leaves	Dysentery	Mastication: Chewing some young leaves	Oral	Swallow the substance obtained from the mastication of the leaves each morning for 3 days	6.58	6.10	2.98
	Diarrhea	Crushing: Crush the leaves and pour into tomato sauce	Oral	Consume the sauce for 3 days	1.88	1.59	3.00
	Fever	Decoction: Boil the leaves in water	Bath or oral	Drink one glass and take shower 3 times a day for 3 days	3.49	2.39	3.00
	Malaria	Maceration: Soaking the leaves in water for 3 days	Oral	Drink one glass 3 times a day for 7 days	28.32	13.26	3.00
	Bee sting	Trituration: Squeezing young leaves	Massage	Apply in the affected body part once and get fine few minutes later	7.12	2.92	3.00
	Intestinal worms	Crushing: Crush and use it to make soup	Oral	Consume the soup for 7 days	8.07	6.37	2.88
	Stomach aches	Trituration: Squeezing young leaves	Oral	Drink one glass three times a day for three days	4.43	2.25	2.74
	Anemia	Maceration: Soaking the leaves in water for 3 days	Oral	Drink one glass three times a day for three days	3.36	1.46	3.00
	Sexual weakness	Trituration: Squeezing young leaves	Oral	Filter, add water or any liquid and drink a glass before having sex	2.42	1.19	3.0
	Snake bite	Chewing: Chew the leaves	Oral	Swallow the juice	2.15	1.33	3.00
	Cold	Decoction: Boiling the leaves in water.	Oral	Drink one glass a day and use it to take shower 3 times a day and for 3 days	1.34	1.33	3.0
Root	Snake bite	Looting: Loot the root, add leaves of *Pseudocedrela kotschyi* (Schweinf.) Harms. or *Ceiba pentandra* (L.) Gaertn	Massage	Apply the obtained product in the affected zone (renewable)	2.82	2.39	3.00
	Swelling of body part	Looting: Loot the root, add some Shea Butter	Massage	Apply the obtained substance in the affected part once a day (renewable)	5.38	2.79	2.95
	Scorpion sting	Looting: Loot the root	Massage	Apply a small amount of the product obtained on the damaged body part once	6.57	1.99	3

Several plant parts of *A. senegalensis* were used for food and for medicinal purposes. Fruits followed by flowers, leaves and seeds were mostly used as food (Fig. 3a). Leaves and roots were the only plant parts used for medicinal purposes (Fig. 3b).

**Fig. 4:**
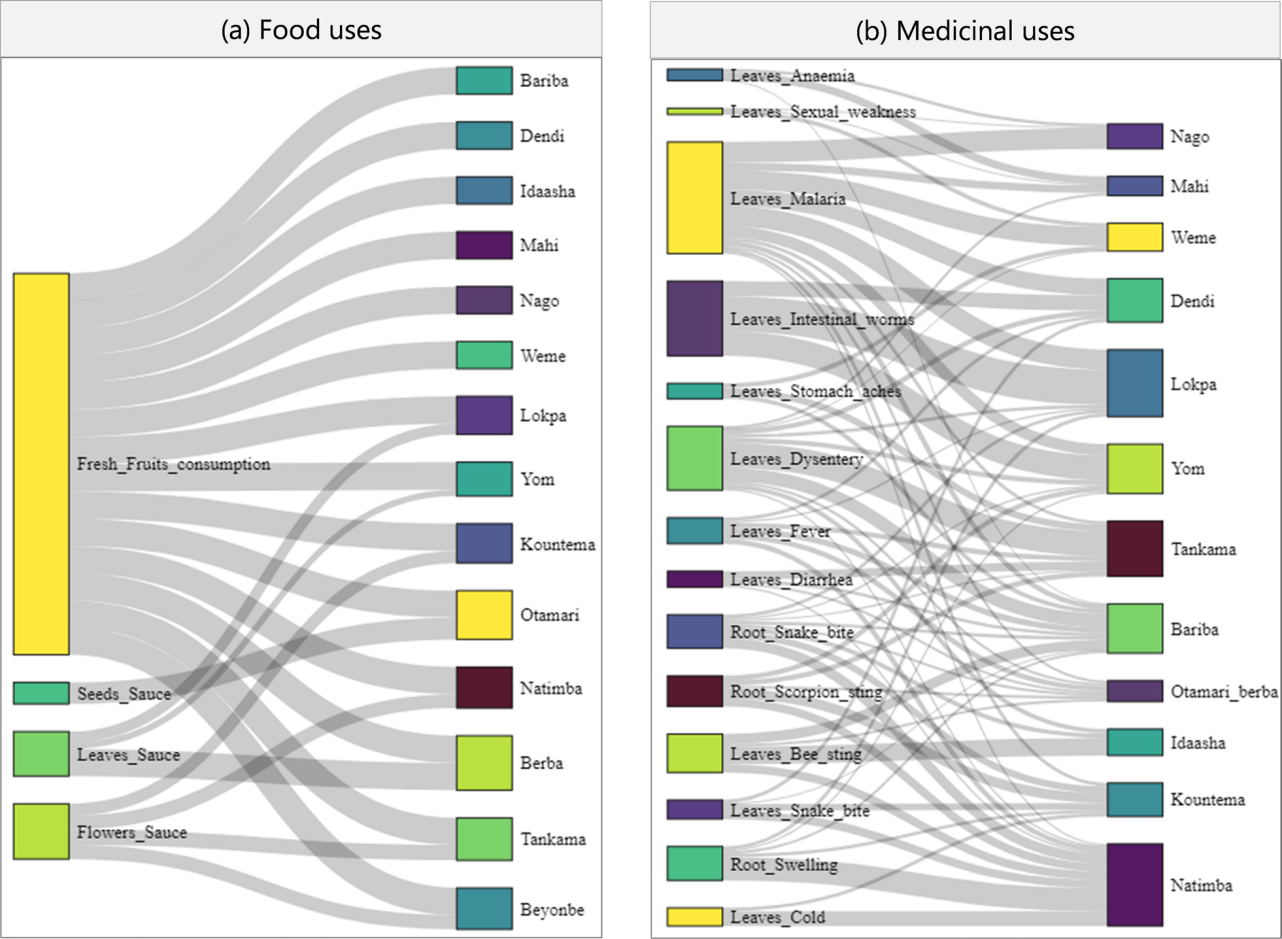
Food (**a**) and medicinal (**b**) uses of *A. senegalensis* and relationship to sociolinguistic groups

Fresh fruits of *A. senegalensis* are commonly eaten by all the sociolinguistics groups surveyed in the study area (Fig. 4a). All informants confirmed that the fresh fruit is tasty and well appreciated. However, the use of flowers, leaves and seeds is different among sociolinguistic groups. For instance, sociolinguistic groups such as Kountema, Natimba, Tankama and Beyonbe use the flowers to make a delicious sticky sauce. The same sticky sauce is rather obtained with leaves by Berba, Lokpa and Yom sociolinguistic groups. In addition, seeds of *A. senegalensis* are especially used as sauce seasoning ingredient by Otamari sociolinguistic group (Fig. 4a).

Sociolinguistic groups use either the roots or the leaves of *A. senegalensis* to heal many diseases (Fig. 4b). The uses of leaves to treat Malaria are common for almost all sociolinguistic groups followed by the uses of the leaves to treat dysentery. Leaves are mostly used for dysentery by Tankama followed by Bariba, Mahi, Weme, Lokpa, Dendi, Yom, Otamari, Berba, Idaasha and Natimba. Sociolinguistic groups such as Lokpa, Yom and Dendi mainly use the leaves for intestinal worms. Concerning the root, it is mostly used to fight snakebites, swelling of body parts (edema) and scorpion stings. Natimba, Kountema, Idaasha, Bariba, Otamari and Berba, Tankama, Yom and Lokpa use mostly the root for snakebite, while Kountema, Natimba, Tankama, Yom, Otamari and Berba use it for scorpion sting (Fig. 4b). Both leaves and roots are, respectively, used for snake bites by different sociolinguistic groups. Roots are used by Natimba, Kountema, Idaasha, Bariba, Otamari and Berba, Tankama, Yom and Lokpa sociolinguistic groups, while leaves are preferred by Natimba, Kountema, Bariba and Dendi.

**Fig. 5:**
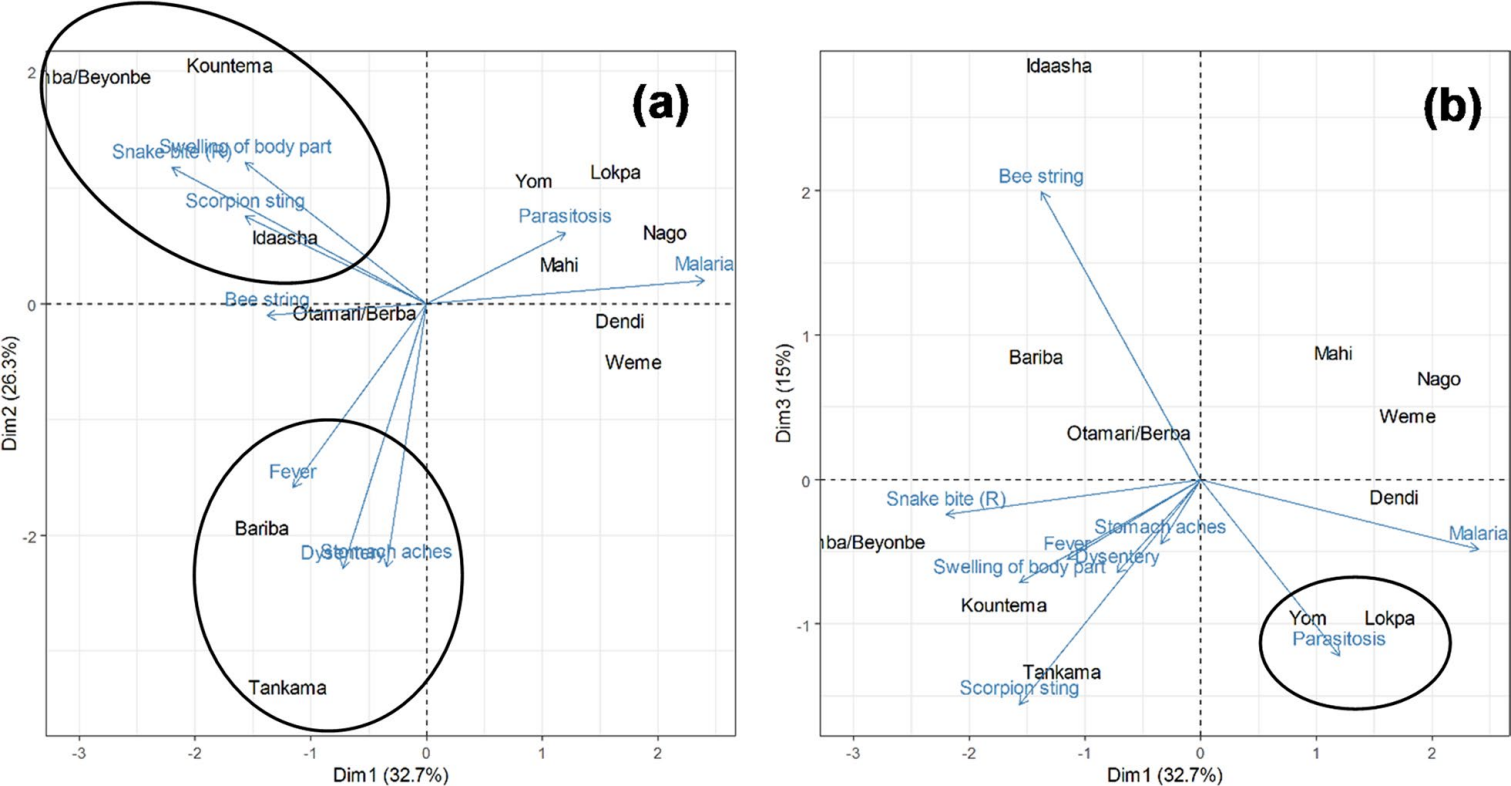
Projection of actual medicinal uses (AU) and sociolinguistic groups on the three first principal components

The PCA revealed that 74% of the initial variation was saved on the first three components (Table 3). The correlation between variables (here the specific medicinal uses) and principal components showed that informants use variously the species as medicine (Fig. 5).

**Table 3:** Correlation between medicinal uses and principal components—significant correlations (those with absolute value greater or equal to 0.5) are highlighted in bold

Medicinal uses	PC1	PC2	PC3
Parasitosis	0.26	0.15	−0.39
Dysentery	−0.16	−**0.56**	−0.21
Fever	−0.25	−0.39	−0.18
Stomach aches	−0.07	−**0.56**	−0.14
Malaria	**0.52**	0.05	−0.16
Bee string	−0.30	−0.03	**0.64**
Swelling of body part	−0.34	0.30	−0.23
Snake bite	−0.48	0.28	−0.08
Scorpion sting	−0.34	0.18	−**0.50**

The use of the species against dysentery is often associated with its use against stomach aches, and fever, and mainly practiced by Bariba and Tankama (see principal component 2). The use of the species against bee string (practiced by Idaasha) is negatively associated with the use of the species against scorpion sting (practiced by Tankama, see principal component 3). Informants from Yom and Lopka sociolinguistic group use the species against parasitosis.

### Factors influencing TK, AU and differences between TK and AU

**Fig. 6:**
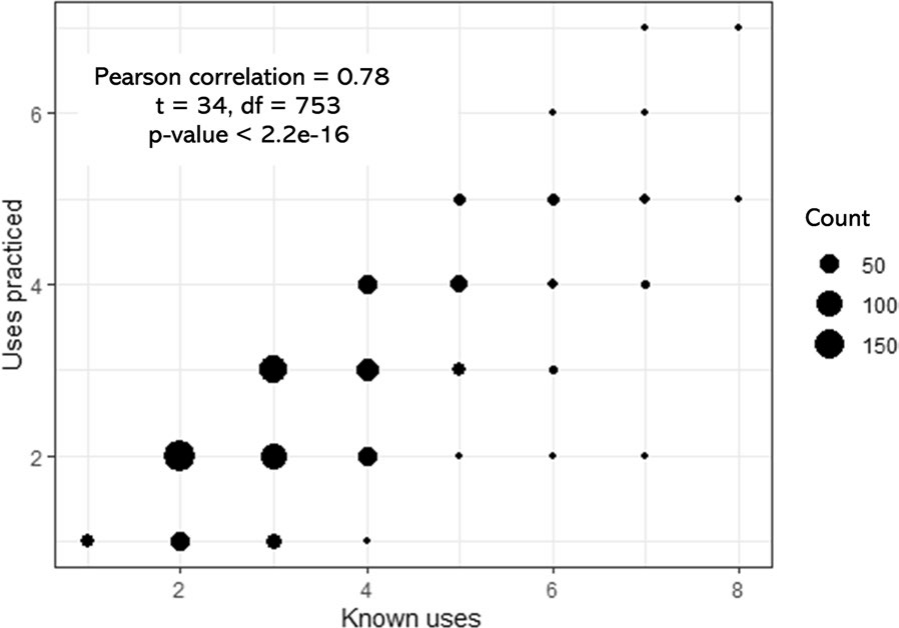
Correlation between known uses and uses practiced. Count stands for the number of overlapping points

The number of known uses varied from 1 to 8 (median value = 3), the number of known uses that were practiced varied from 1 to 7 (median value = 2), and the gap between the two varied from 0 to 7 (median value = 0). There was a significant positive correlation between the number of known uses and the number of uses that were practiced (Pearson correlation = 0.78, *t* = 34, df = 753, *p*-value < 2.2e−16) (Fig. 6).

From all candidate models to explain variation in TK, AU and differences between TK and AU, the most parsimonious included main activity and sociolinguistic groups for TK and AU (Table 4). Both factors explained about 36% and 53% of variation in TK and AU, respectively. The difference between TK and AU was  mainly related to sociolinguistic group (Table 4).

**Table 4:** Summary of model selection among candidate models for TK_Total_, AU_Total_, AU_Food_, AU_Medicinal_, and TK_Total_—AU_Total_

Candidate models	TK_Total_		AU_Total_		AU_Food_		AU_Medicinal_		TK_Total_–AU_Total_	
	AICc	∆AICc	AICc	∆AICc	AICc	∆AICc	AICc	∆AICc	AICc	∆AICc
SLG	–	–	–	–	1662.4	0	–	–	1519.1	0
SLG + Sex	–	–	–	–	1663.0	0.6	–	–	1519.1	0
SLG + SPG	2549.7	0	2265.6	0	–	–	1852.9	0	–	–
SLG + SPG + Sex	2550.9	1.2	2266.8	1.2	–	–	–	–	1519.1	0
SLG + Age + Sex	–	–	–	–	1666.6	4.2	1854.8	1.8	–	–
SLG + Age + SPG + Sex	2554.5	4.8	2270.6	5.0	–	–	1857.0	4.1	1523.4	3.5
SLG + Age + Sex + Age: Sex	–	–	–	–	1670.5	8.1	–	–	–	–
SLG + Age + SPG + Sex + Age: Sex	2558.4	8.7	2274.2	8.6	1674.5	12.1	1859.9	6.9	1526.8	6.9
SLG + Age + SPG + Sex + Age: Sex + Sex: SLG	2566.1	16.4	2284.5	18.8	1693.3	30.9	1865.4	12.5	1534.9	14.9
SLG + Age + SPG + Sex + Age: Sex + Age: SLG + Sex: SLG	2595.8	46.1	2318.5	52.8	1734.3	71.9	1895.1	42.2	1549.1	29.1
Goodness of fit test	0.999		0.999		0.998		0.999		0.999	
Model significance test	< 0.001		< 0.001		< 0.001		< 0.001		< 0.001	
Nagelkerke *R*^2^ (%)	35.74		42.23		32.61		34.98		28.02	

*TK*_*Total*_ theoretical knowledge, *AU*_*Total*_ actual uses, *AU*_*Food*_ actual uses for food use-category, *AU*_*Medicinal*_ actual uses for medicinal use-category, *SPG* socio-professional group, *SLG* sociolinguistic group

Actual food use-value differed significantly among sociolinguistic groups, whereas in addition to sociolinguistic groups, main activity also affected the medicinal use-value. Sociolinguistic group explained 33% of the variation in food use-value, whereas both sociolinguistic group and main activity explained 35% of medicinal use-value (Table 4).

**Fig. 7:**
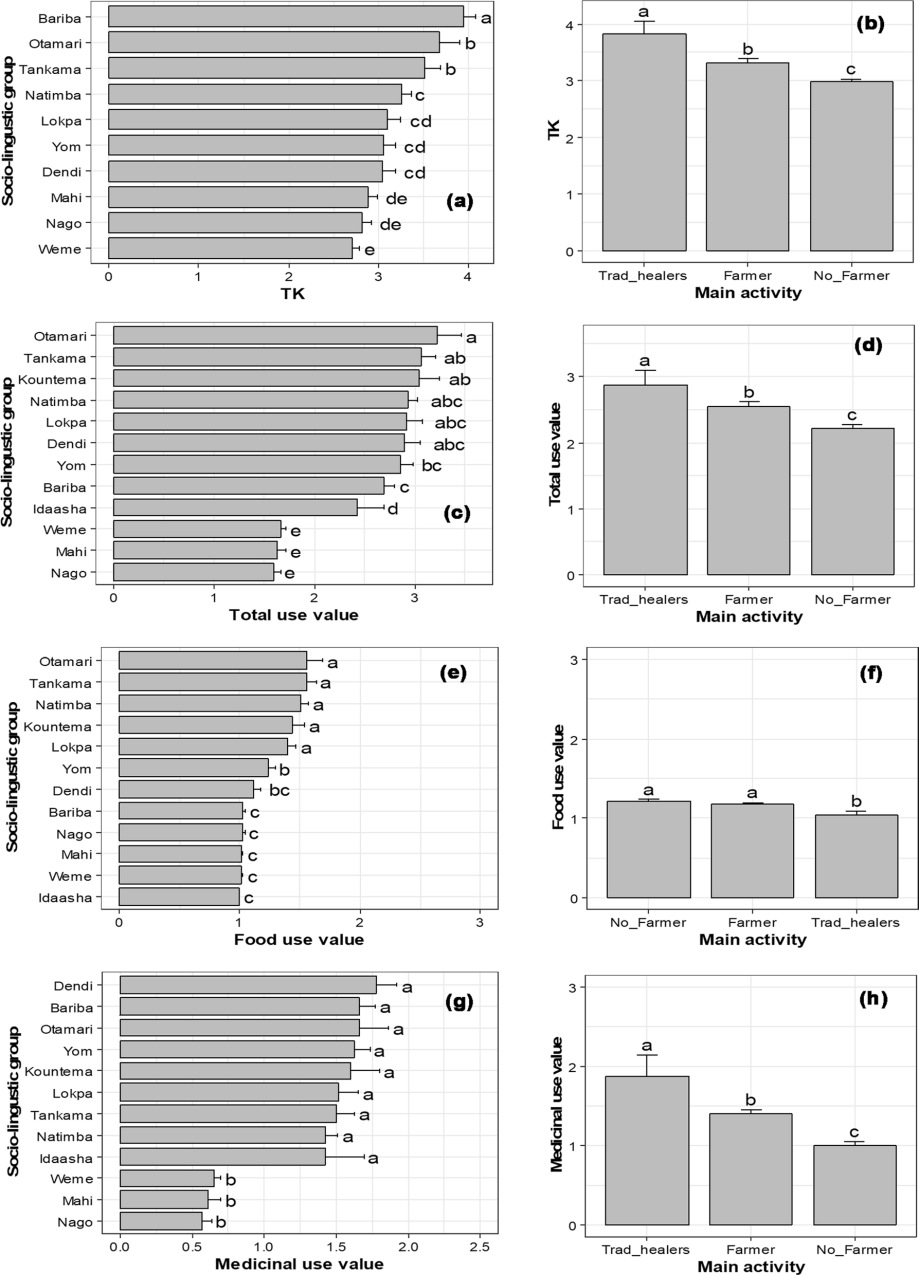
Variation in TK_Total_, AU_Total_, AU_Food_, AU_Medicinal_ of *A. senegalensis* according to sociolinguistic group and main activity. Bars with different letters indicate significant differences

TK was higher for Bariba sociolinguistic group and lower for Wémènou (Fig. 7a). Traditional healers had the highest TK, and the non-farmers had the lowest TK (Fig. 7b). Informants from the Otamari sociolinguistic group had the highest total use-value followed by Tankama and Kountema, while Wémènou, Mahi and Nago had the lowest total use-value (Fig. 7c). Like TK, traditional healers had the highest total use-value and the non-farmers the lowest total use-value (Fig. 7d). Informants from Otamari, Tankama, Natimba, Koutema and Lokpa sociolinguistic groups had the highest food use-value, whereas informants from Bariba, Nago, Mahi, Wémènou and Idaasha sociolinguistic groups had the lowest food use-value (Fig. 7e). The food use-value was not different between farmers and non-farmers but the knowledge of traditional healers about food use was less than the one of the two above-mentioned socio-professional groups (Fig. 7f). Informants from sociolinguistic groups Dendi, Bariba, Otamari, Yom, Kountema, Lokpa, Tankama, Natimba and Idaasha had the highest medicinal use-value, whereas informants from sociolinguistic groups Wémènou, Mahi and Nago had the lowest medicinal use-value (Fig. 7g). With respect to the main activity, traditional healers had the highest medicinal use-value, followed by farmers, and finally non-farmer’s informants (Fig. 7h).

**Fig. 8:**
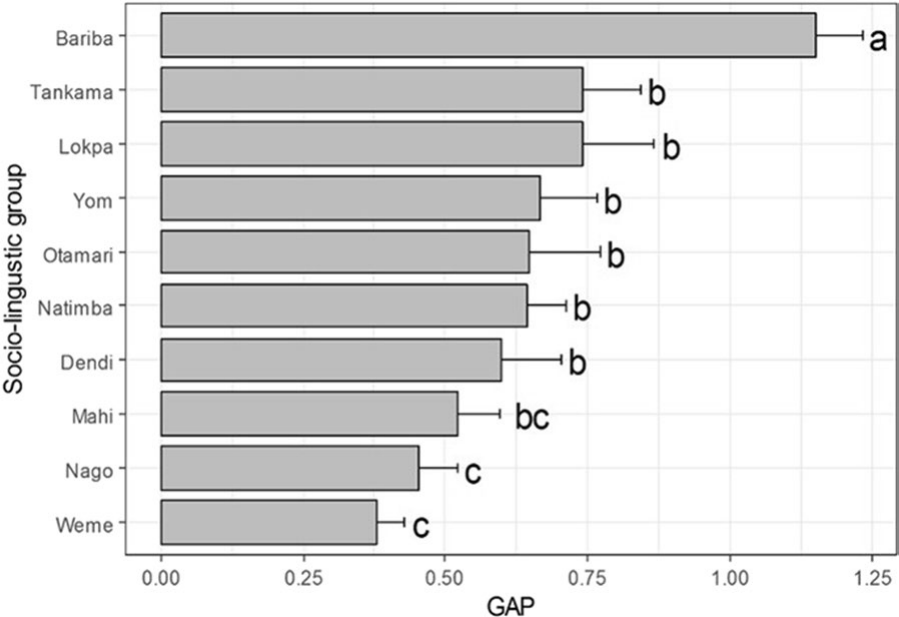
Difference between theoretical traditional knowledge and actual uses according to sociolinguistic group. Sociolinguistic groups with different letters are significantly different

Informants from the sociolinguistic group Bariba had the highest gaps between TK_Total_ and AU_Total_ (Fig. 8). They were followed by informants belonging to Tankama, Lokpa, Yom, Otamari, Natimba, and Dendi sociolinguistic groups, whereas Nago and Wémènou had the lowest difference between TK and AU (Fig. 8).

## Discussion

### Diversity of uses of *A. senegalensis*

Our findings revealed that *A. senegalensis* is a well-known and used species in Benin. Fruits and flowers are the most used plant parts for food purposes, while leaves and roots are mostly used for medicinal purposes. All the informants consume and appreciate the fruits. The ripen fruits constitute a real food for the local population. They argued that fruits have good flavor and serve to calm hunger especially during field work, while the aromatic flowers are used to flavor sauce. In Burkina Faso, [33] also reported the fruits and the flowers as highly used parts of *A. senegalensis* for food by local communities.

Other plant parts of the species have been cited by informants in the treatment of various diseases. Leaves and roots are the most cited plant parts for medicinal uses. Local populations hold a great diversity of ethnobotanical knowledge on *A. senegalensis* and use the species as food and for health care as supported by previous studies in Nigeria [16, 34]. In Benin, leaves and roots are known to treat malaria, dysentery, snake bite, swelling of body parts, among others, but the relative frequency of citation of their actual uses is low for most plant parts (from 1.34 to 28.32%). These low values indicate that knowledge on the uses of the species is unequally distributed among local people, but also suggest low consensus on many medicinal uses. The use of the leaves of *A. senegalensis* for the treatment of malaria had the highest RFC in our study. This use has also been noted as common use in traditional medicine in Nigeria and the Republic of Guinea (Additional file 1).

While gathering traditional knowledge on the use of plants is necessary, ethnopharmacological investigations are essential to confirm traditional uses and pave the way for drug discovery. The use of leaves of *A. senegalensis* to treat malaria is supported by several ethnopharmacological studies. For example, Ajaiyeoba et al. [35] demonstrated that the methanol extract of *A. senegalensis* has a better antimalarial activity against *Plasmodium berghei* Vincke & Lips than the standard reference drug chloroquine diphosphate which had a 96.2% chemo suppression activity. In Cameroon, the fractions efficacy of *A. senegalensis* leaf extract on immature stage development of malarial and filarial mosquito vectors were also evaluated in laboratory. The leaf extract of *A. senegalensis* was toxic on immature stage of *Anopheles gambiae* Giles and *Culex quinquefasciatus* Say*.* The N-hexane and chloroform fractions extract from the species were recommended to be used for immature mosquito vectors control [36]. Many other studies have confirmed the use of *A. senegalensis* in the treatment of malaria [37, 38]*.* In addition, the antivenomous activity *of A. senegalensis* has been corroborated with ethnopharmacological evidence. For example, the methanol extract of the root bark of *A. senegalensis* tested against cobra (*Naja nigricollis nigricollis* Welch) venom in rats resulted in significant reduction of the induced hyperthermia and directly detoxified the snake venom [39]. Furthermore, Emmanuel et al. [40] examined the effect of a fraction of *A. senegalensis* leaf methanol extract on *Echis ocellatus* Stemmler venom. The extract neutralized lethal toxicity induced by *E. ocellatus* venom [40]. These examples show that *A. senegalensis* is an interesting medicinal shrub species in African cultures which deserve particular attention for its efficient uses and effective conservation.

### Differences between TK and AU of *Annona senegalensis*, and influencing factors

TK and AU are different information often gathered during ethnobotanical investigations, but sometimes used interchangeably. Not distinguishing between the two may lead to confusion and mislead decision-making, for e.g., in species assessment, valorization, conservation, and sustainable management.

Consistent with several previous findings (e.g., Reyes-García et al.) [8] and our first hypothesis, we found a positive correlation between TK and AU indicating that the more knowledgeable is the informant, the more frequent he/she actually uses the species. As predicted, we also found a significant difference between the use-value calculated based on TK and the one based on AU for *A. senegalensis*, and furthermore a positive correlation between TK and the difference between TK and AU. The latter indicates that the more knowledgeable is the informant, the higher the difference between theoretical knowledge on uses and actual uses. Several reasons may explain the difference between TK and AU. First, the difference might be attributed to modernity which leads certain people with greater knowledge on medicinal plants to prefer modern medicine over indigenous substitute plants [41]. Second, the more knowledgeable people on *A. senegalensis*, certainly also know several other species that have redundant uses [42] with *A. senegalensis*, and perhaps more effective than *A. senegalensis*. Therefore, the knowledge they have on *A. senegalensis* is not valued in practice, thus making the actual use-value to be lower than the TK.

Previous studies suggested that the gap between TK an AU can be due to differences in socioeconomic and demographic contexts (e.g., Reyes-García et al.) [8]. At the informant level, our results showed that the gap between TK an AU varied only according to the sociolinguistic group, not age and sex. Specifically, the gap was higher for Bariba, Tankama and Lokpa and lower for Nago and Wemènou. Bariba sociolinguistic group were also the ones who had the highest value of TK, indicating that they are the most knowledgeable on the species. The observed variation in the gap among sociolinguistic groups could be explained by the unequal transmission of knowledge within sociolinguistic groups. Indeed, local knowledge on plant species is accumulated over the time and are transmitted throughout generations [43, 44] with different patterns according to sociolinguistic groups. Indigenous people who are the first users of plants usually have an immense knowledge on them. In addition, the virtues of plants are often ancestral knowledge and specific to the context and history of each community [45, 46]. Thus, this result suggests that sociolinguistic groups Bariba and Tankama who have more TK on *A. senegalensis* and do not practice enough might just have heard about the uses of the species and did not have the opportunity to practice because the species is becoming less abundant in their area compared to other species that probably have similar virtues. Some authors also reported that local populations know more on plants than they practice in reality [13, 47, 48]. Yet, there are several cases where TK and AU are used interchangeably to assess the use-value of species. This result illustrates that assessing the use-value of a species based solely on the TK could lead to an overestimation of the species use-value, and consequently its interpretation, for e.g., the pressure exerted on a species for  its use by human. Indeed, some studies have used species use-value as a metric of human pressure [12]. Furthermore, to test the ecological appearance hypothesis (species that are more apparent in a given area  are more used), the calculation of species use-value should consider only the current use [9].

As such, both TK and AU are relevant in describing the usefulness of a species, but differentiating TK and AU is necessary when estimating availability of resources in a given community and when planning for improving locals’ livelihoods. This is crucial because traditional knowledge on food and medicinal uses of plants is becoming more important in food and health industries. Therefore, distinguishing between TK and AU can then help in avoiding confusion while working to discover new drugs.

### Socio-demographic factors influencing TK and AU of *A. senegalens*is

Our findings revealed that mainly sociolinguistic and socio-professional groups significantly influence the theoretical knowledge and actual uses of *A. senegalensis.* The food use-value also varies among sociolinguistic groups. While some sociolinguistic groups use the flowers and/or the seeds to prepare sauce (Beyonbe, Tankama, Natimba, Kountema and Fon), others (Berba, yom, Lokpa, Wemènou and Mahi) prefer the leaves. This difference could be explained on the one hand by the cultural differences between sociolinguistic groups and on the other hand by the difference in culinary attitude which mostly vary across localities. Similar variation in food use between sociolinguistic groups has been reported on the uses of some fruit tree species such as *Parkia biglobosa* (Jacq.) R. Br. ex G. Don [49] and *Adansonia digitata* L. [50]. Our results showed the same variation in medicinal uses of the species. Sociolinguistic groups Dendi, Bariba, Otamari, Yom, Kountema, Lokpa, Tankama, Natimba and Idaasha had the higher medicinal use-value. Bariba sociolinguistic group had the higher theoretical knowledge, while Otamari had the higher actual uses. Sociolinguistic group is one of the widely reported factors that drive difference in the use-value of species, either plants or animals [51, 52]. We also found that the main activity influences on the medicinal, food and overall use-value of *A. senegalensis*. Specifically, traditional healers had the higher medicinal use-value, which is likely related to their historical link with nature and traditional practices, unlike non-professional healers like farmers and non-farmers.

## Conclusions

This paper used the case-study of *A. senegalensis * in Benin to illustrate important difference between traditional theoretical knowledge and actual uses. We showed that there is a significant difference between the use-value calculated based on theoretical knowledge and the one calculated based on actual uses. Consequently, species assessment based on either TK or AU might led to different conclusions, and not distinguishing between the two might led to erroneous interpretations. Furthermore, the study provides interesting insights on the food and medicinal uses of an important African annonaceae. There is a wide consensus on the use of the fruit and flowers for human food. Also, the most common medicinal uses of the species leaves to treat malaria in the study area is supported by ethnopharmacological studies on the ethanol extract of the leaves. Despite these uses and the species importance for local people, *A. senegalensis* is still at the infant stages of domestication. Engaging effective domestication of the species can contribute to diversify the source of income for local communities and thereby enhance their resilience to various pressures. 

## Supplementary Information

**Additional file 1**. Common uses of *A. senegalensis* reported in other african countries.

## Data Availability

The datasets used and/or analyzed during the current study are available from the corresponding author on reasonable request.

## Abbreviations

TK: Theoretical knowledge
AU: Actual uses
AU_Food_: Actual uses for food use-category
AU_Medicinal_: Actual uses for medicinal use-category
SPG: Socio-professional group
SLG: Sociolinguistic group
TUV: Total use-value
INSAE: Institut National de la Statistique et de l’Analyse Economique
