# Supplementary material for: Food and medicinal uses of Annona senegalensis Pers.: a country-wide assessment of traditional theoretical knowledge and actual uses in Benin, West Africa
Source: J Ethnobiol Ethnomed. 2022 Mar 4;18:10. doi: 10.1186/s13002-022-00510-2 (PMC8894562; doi:10.1186/s13002-022-00510-2)
Supplement: Supplementary file 1 — Additional file 1. Common uses of A. senegalensis reported in other african countries. [file 13002_2022_510_MOESM1_ESM.docx]

**Supplementary file 1. Common uses of *A. senegalensis* reported in other countries**

| Disease/ Disorders | Plant parts | Countries | Reference |
| --- | --- | --- | --- |
| Malaria | Leaves | Republic of Guinea | [20] |
|  | Leaves | Nigeria | [4] |
|  | Leaves | Cameroon | [19] |
| Menstrual disorder, hemorrhoids, Hernia, | Root | Angola | [9] |
| Leg pain, abdominal pain, Worms, | Leaves |  |  |
| Epilepsy and convulsions | Root bark | Burkina Faso | [12] |
| Epilepsy, snake bite, gastritis, male sexual  Impotence | Root and Leaves | Cameroun | [18, 11, 6] |
| Diabetes | Leaves,  Bark and seed | Congo-Brazaville | [2] |
| Sickle-cell anemia | Leaves | Democratic Republic of Congo | [5] |
| Asthma, cough | Leaves | Ivory coast | [22] |
| Mental illnesses | Root | Mali | [15] |
| Smallpox, liver cancer, leukemia, yellow fever, tuberculosis. | Leaves | Nigeria | [16, 21, 3, 17, 1] |
| Skin cancer, snake bites, Hernia | Stem | Nigeria | [7, 1] |
| Infectious diseases, including sexually transmitted diseases. | Root bark | Republic of Guinea | [13] |
| Maternal health-related problems, infertility, improved sexual performance, pains during pregnancy, fever, or oedema. | Root | South Africa | [10, 14] |
| Oedema, stomach problems, or easy labour | Bark | South Africa | [14] |
| Alleviation of stomach problems in babies. | Leaves |  |  |
| High blood pressure | Root | Tchad | [8] |

**References**

1. Abubakar MS, Musa AM, Ahmed A, Hussaini IM. The perception and practice of traditional medicine in the treatment of cancers and inflammations by the Hausa and Fulani tribes of Northern Nigeria. J. Ethnopharmacol. 2007 ;111(3):625-9.
2. Ahombo G, Ampa R, Diatewa M, Mpati J, Abena A, Ouamba J. Investigating on related diabetes therapeutic plants used in traditional medicine at Brazzaville. J Med Pl Res. 2012; 6(44):5630-5639.
3. Aiyeloja A, Bello O. Ethnobotanical potentials of common herbs in Nigeria: A case study of Enugu state. Educ. Res. Rev. 2006; 1: 16-22.
4. Ajaiyeoba E, Falade M, Ogbole O, Okpako L, Akinboye DO. In Vivo Antimalarial and Cytotoxic Properties of Annona SenegalensisExtract. AJTCAM. 2005; 3: 137-141.
5. Bongo G, Inkoto C, Masengo C, Tshiama C, Lengbiye E, Djolu R, Mutwale K, Kabamba K, Mbemba T, Tshilanda D, Mpiana PT, Ngbolua KN. Assessment of antisickling, antioxidant and antibacterial activities of some Congolese Taxa: Aframomum alboviolaceum (Ridley) K. Schum, Annona senegalensis Pers. and Mondia Whitei (Hook. f.) Skeels. AJLM. 2017;2(4): 52-59. DOI: 10.11648/j.ajlm.20170204.13
6. Bum EN, Taiwe GS, Moto FC, Ngoupaye GT, Vougat RR, Sakoue VD, Gwa C, Ayissi ER, Dong C, Rakotonirina A, Rakotonirina SV. Antiepileptic medicinal plants used in traditional medicine to treat epilepsy. InClinical and genetic aspects of epilepsy 2011. IntechOpen.
7. Dambatta S, Aliyu B. A Survey of Major Ethno Pharmacognosy and Phytochemistry medicinal plants of Kano North, Nigeria, their Knowledge and Uses by Traditional Healers, Bayero J Pure Applied Sci. 2011; 4(2):28-34.
8. Dongock DN, Bonyo AL, Mapongmestem PM, Bayegone E. Ethnobotanical and phytochemical study of medicinal plants used in the treatment of cardiovascular diseases in Moundou (Tchad). Int. j. biol. chem. 2018;12(1):203-16.
9. Göhre A, Toto-Nienguesse ÁB, Futuro M, Neinhuis C, Lautenschläger T. Plants from disturbed savannah vegetation and their usage by Bakongo tribes in Uíge, Northern Angola. J. Ethnobiol Ethnomed. 2016 Dec;12(1):1-28.
10. ILBOUDO S, OUEDRAOGO GG, OUEDRAOGO S, GUISSOU IP. Phytochemical, acute and subacute toxicity studies of Annona senegalensis Pers.(Annonaceae) root wood extracts. Afr. J. Biochem. Res.2019 ;13(4) :44-55.
11. Jiofack, T., Fokunang, C., Guedje, N., Kemeuze, V., Fongnzossie, E., Nkongmeneck, B. A., ... & Tsabang, N. (2009). Ethnobotanical uses of some plants of two ethnoecological regions of Cameroon Afr. J. Pharmacy Pharmacol., *3*(13), 664-684.
12. Konate A, Sawadogo WR, Dubruc F, Caillard O, Ouedraogo M, Guissou IP. Phytochemical and Anticonvulsant Properties of" Annona senegalensis" Pers.(Annonaceae), Plant Used in Burkina Folk Medicine to Treat Epilepsy and Convulsions. Br. J. Pharmacol. 2012 ;3(5):245-50.
13. Magassouba FB, Diallo A, Kouyaté M, Mara F, Mara O, Bangoura O, Camara A, Traoré S, Diallo AK, Zaoro M, Lamah K. Corrigendum to “Ethnobotanical survey and antibacterial activity of some plants used in Guinean traditional medicine” J. Ethnopharmacol. 2010 21;128(3):705-8.
14. 14. Mahwasane ST, Middleton L, Boaduo N. An ethnobotanical survey of indigenous knowledge on medicinal plants used by the traditional healers of the Lwamondo area, Limpopo province, South Africa. S. Afr. J. Bot. 2013 Sep 1; 88:69-75
15. Mounkoro PP, Togola A, de Jong J, Diallo D, Paulsen BS, van’t Klooster C. Ethnobotanical survey of plants used by traditional health practitioners for treatment of schizophrenia Spectrum disorders in Bandiagara, mali, West Africa. J. Herb. Med. 2020 Dec 1; 24:100402.
16. Mustapha A, Owuna G, Uthman I. Plant Remedies Practiced by Keffi People in the Management of Dermatosis, J. Med. Pl. Stu. 2013; 1(5):112-118.
17. Mustapha AA, Owuna G, Uthman IHI. Plant remedies practiced by Keffi people in the management of dermatosis. J. Med. Plant Res.2013; 1: 112-118.
18. Noumi E, Safiatou M. Some Investigations on the Traditional Pharmacopoeia about Venomous Bites and Stings from Scorpions, Snakes As A and Spiders in the Hina Subdivision, Far North, Cameroon, British J Pharm Res. 2015, 344-358.
19. Suleiman M, Mamman M, Igomu EE, Muhammad Y, Talba AM. Evaluation of analgesic and anti-inflammatory effects of the crude methanol extract of the stem-bark of Annona senegalensis Pers.Int. j. med. aromat. plants. 2014; 4: 88-96.
20. Traore MS, Baldé MA, Diallo MS, Baldé ES, Diané S, Camara A, Diallo A, Balde A, Keïta A, Keita SM, Oularé K. Ethnobotanical survey on medicinal plants used by Guinean traditional healers in the treatment of malaria. J. Ethnopharmacol.. 2013 Dec 12;150(3):1145-53.
21. Yakubu, O. F., Metibemu, D. S., Adelani, I. B., Adesina, G. O., Edokwe, C. B., Oseha, O. E., & Adebayo, A. H. (2020). Annona senegalensis extract demonstrates anticancer properties in N-diethylnitrosamine-induced hepatocellular carcinoma in male Wistar ratsBiomed. Pharmacother, *131*, 110786.
22. Yeo D, Dinica R, Yapi HF, Furdui B, Praisler M, Djaman AJ, N'Guessan JD. Evaluation of the anti-inflammatory activity and phytochemical screening of Annona senegalensis leaves. Therapie. 2011 ;66(1):73-80.
